# Nitric Oxide Levels and Total Antioxidant Capacity in The Seminal Plasma of Infertile Smoking Men

**DOI:** 10.22074/cellj.2015.519

**Published:** 2015-04-08

**Authors:** Yousefreza Yousefniapasha, Gholamali Jorsaraei, Maryam Gholinezhadchari, Soleiman Mahjoub, Mahmoud Hajiahmadi, Mehrdad Farsi

**Affiliations:** 1 Fatemeh-zahra Infertility and Reproductive Health Research Center, Babol University of Medical Sciences, Babol, Iran; 2 Department of Biochemistry and Biophysics, Faculty of Medicine, Babol University of Medical Sciences, Babol, Iran; 3 Infectious Diseases and Tropical Medicine Research Center, Babol University of Medical Sciences, Babol, Iran

**Keywords:** Smoking, Nitric Oxide, TAC, Male Infertility

## Abstract

**Objective:**

Cigarette is a rich source of oxidants and reactive nitrogen species. Nitric oxide (NO) in high concentration has deleterious effects on human sperm function. Antioxidant defense system in seminal plasma protects spermatozoa from the attack of reactive oxygen metabolites. The purpose of this study was to determine the correlation between cigarette smoking with the NO levels and the total antioxidant capacity (TAC) of the seminal plasma in infertile smoker men and to compare severity of oxidative stress (OS) in them with fertile and infertile non-smoking men.

**Materials and Methods:**

In this cross sectional study, a total of 95 male participants attended the Infertility Clinic of the Fatehmeh-zahra Hospital in Babol, Mazandaran Province, Iran, between 2010 and 2011. They were divided into three groups: I. fertile non-smokers (F.ns; n=32), II. infertile non-smokers (IF.ns; n=30) and III. infertile smokers (IF.s; n=33) according to semen analysis World Health Organization guidelines (WHO, 2001) and smoking data. TAC concentration and NO levels of seminal plasma were measured using the ferric reducing ability of plasma (FRAP) method and the Griess reagent, respectively.

**Results:**

Standard sperm parameters were significantly higher in the fertile group than those in the infertile groups, but these differences between the IF.ns and IF.s were not statistically significant. The mean TAC in the seminal plasma was higher in the F.ns>IF.ns>IF.s, respectively, but these differences were not statistically significant among three groups (p= 0.096). In contrast, the mean NO level in the seminal plasma was lower in the F.ns<IF.ns< IF.s, respectively. These differences were statistically significant among the three groups (p= 0.018).

**Conclusion:**

It argued that the increased NO levels associated with smoking might exceed the capacity of antioxidant defense system, leading to increased oxidative damage of seminal plasma and decreased fertility in men.

## Introduction

Cigarettes contain a wide category of xenobiotics, including oxygen free radicals and reactive nitrogen species ( RNS ) (1). It has been shown that the enlarged production of reactive oxygen species ( ROS ) related to smoking could increase the capacity of oxidant defense system, resulting in redoubled aerobic injury of seminal plasma (2,3). Smoking-tobacco contains nearly 4000 harmful compounds like alkaloids, nitrosamines, nicotine and hydroxycotinine that many of these substances generate ROS and RNS (4). Smoking has been shown to be associated with a 48 and 107% increase in seminal leukocyte and ROS concentrations, respectively (5). Among the body cells, spermatozoa due to small amounts of cytoplasm enzymes are very sensitive to oxidative damages and are unable to repair them (6). On the other hand, the plasma membrane of spermatozoa possesses high polyunsaturated fatty acids ( PUFA ), which make them susceptible to ROS by inducing lipid peroxidation ( LPO ) (6,7). A physiological level of ROS during normal aerobic metabolism in spermatozoa is essential for maturation, capacitation, acrosomal reactions, and spermatozoan-oocyte fusion as well as for the general function or viability of spermatozoa (8,9). However, the production of excessive levels of ROS can be potentially toxic for sperm function (6,7). High levels of ROS may also affect the sperm axoneme, inhibit mitochondrial function and disturb the synthesis of nucleic acids ( DNA and RNA ) and proteins (10). ROS decreases motility of spermatozoa due to damages to the flagellum and axonemal structures of their tails (11). 

Furthermore, it has been shown that ROS can damage DNA by causing base modification, DNA strand breaks, DNA cross-links, and chromosomal rearrangements (9,12). Immature or abnormal spermatozoa and leukocytes are two major sources of production of free radicals in semen (2,6). The most common ROS with significant effects in reproductive biology are as follows: superoxide anion ( O_2_ ), hydrogen peroxide ( H_2_O_2_), peroxyl radicals ( ROO^-^), and also the highly reactive hydroxyl radicals ( OH^-^) (7). The nitrogen species derived from nitric oxide ( NO• ) and peroxynitrite anion ( ONOO^-^) additionally seem to play a significant role within the reproduction and fertilization (7). NO is an oxygen free radical generated from the oxidation of L-arginine to L-citrulline by three isoforms of nicotinamide adenine dinucleotide phosphate ( NADPH )-dependent NO synthases ( NOS ) (13). However, it has been additionally reported that formation of NO and ONOOare caused by use of D-arginine in class spermatozoa mistreatment, cannot be a substrate for NOS. Under these conditions, NO production may be induced nonenzymatically through a H_2_O_2_mediated attack on arginine (14) and also shows cytotoxic effects that are mediated indirectly through its interaction with O_2_and formation of ONOO^-^, which decomposes to form OH^-^and NO_2_, respectively (15). NO features a twin impact on sperm performs, so under physiological conditions, NO plays a vital role in normal sperm production and motility. Low NO levels have been shown to increase sperm motility (16), capacitation (17) and zonapellucida spermbinding protein (18), whereas a number of studies have shown a negative effect of high NO levels in seminal plasma on human sperm motility (13,19). Overall, these studies have suggested a relevant role of NO within the pathophysiology of spermatozoa. The oxidative damage induced by free radicals is commonly decreased by a mix of biological inhibitor systems as total antioxidant capacity ( TAC ) that contains both enzymatic and non-enzymatic reactions. Human seminal plasma comprises the following three main enzymatic antioxidants: superoxide dismutase ( SOD ), catalase, and glutathione peroxidase/glutathione reductase ( GPX/GRD ). Furthermore, there is a wide range of non-enzymatic antioxidants as follows: ascorbate, urate, vitamin E, pyruvate, glutathione, albumin, vitamin A, ubiquitol, taurine, and hypotaurine (11). A ntioxidants are the major defense system against oxidative stress ( OS ) induced by free radicals. However, OS is the result of an imbalance between the generation of ROS and the antioxidant system that would have harmful effects on human spermatozoa (6,7,9). This study aimed to estimate the effects of cigarette smoking on the increased NO levels and decreased TAC in seminal plasma and to compare severity of OS in the infertile smoker men with fertile and infertile non-smoking men. 

## Materials and Methods

### Sample collection and semen analysis

In this cross sectional study, a total of 95 semen samples were provided by donors with the age range 24-38 years attending the Infertility Clinic of the Fatemeh-zahra Hospital in Babol, Mazandaran Province, Iran, between 2010 and 2011. The individuals with a significant medical history, signs of defective and rogenisation, abnormal testicular examinations, chromosomal disorders related to a fertility disorder, cryptorchidism, vasectomy, endocrine disorders, leukocytospermia, alcohol consumption and treated with antioxidant supplements were excluded from this study. The semen samples were collected into sterile container after anabstinence period of 2-3 days at the *in vitro* fertilization ( IVF ) center. Specimens were allowed to liquefy at 37˚C for 30 minutes. The routing analysis of sperm parameters including volume, count, total count and motility was performed within 1 hour according to World Health Organization guidelines ( WHO, 2001 ) using microscopic examination (20). Sperm morphology <14% were considered abnormal according to Kruger’s criteria (21). Then, based on demographic information and medical history of the participants such as age and other disease at the time of sampling/ spermogeram analysis, the subjects were classified into three groups including: i. fertile non-smokers ( F.ns; n=32 ) who had normal semen, ii. infertile non-smokers ( IF.ns; n=30 ) and iii. infertile smokers ( IF.s; n=33 ). Both IF.s and IF.ns groups had a history of primary infertility at least for 1 year and showed abnormal semen analysis, while smokers had consumed 10-20 cigarettes per a day for at least 2 years. 

## Measurement of TAC using the ferric reducing ability of plasma (FRAP) method

The semen samples were centrifuged at 12000 g for 7 minutes and stored at –20˚C until analyt sis. Seminal plasma was thawed at room temperature and diluted 1:10 v/v in distilled water. TAC was then evaluated using the FRAP method according to the method described by Benzie and Strain (22) with slight modifications. This method measures the ability of antioxidants of a sample to reduce ferric-tripyridyltriazine ( Fe^3+^-TPTZ ) to a ferrous form ( Fe^2+^). In brief, the working FRAP reagent was prepared by mixing 300 mmol/L acetate buffer pH=3.6 ( 3.1 g sodium acetate×3H_2_O ( Merck, Germany ) and 16 ml acetic acid in 1000 ml buffer solution) with 10 mmol/L 2,4,6,-tripyridyls-triazine ( TPTZ, Merck, Germany ), 40 mmol/l Hydrochloric acid ( HCl; Merck, Germany ), and 20 mmol/l FeCl_3_×6H_2_O ( Sigma, America ) in a 10:1:1 ratio, just before use. Then, 1.5 mL of the working FRAP reagent was aliquoted into a glass tube and warmed to 37˚C for 5 minutes. Subse˚ quently, 50 μL of plasma, 50 μL of distilled water ( reagent-free ), as well as 50 μL of each of the standard solutions ( FeSO_4_.7H_2_O; 1000, 500, 250, and 125 μM, Sigma, America ) were added to 1.5 mL FRAP reagent and heated in 37˚C for 10 ming utes. Absorbance was measured at 593 nm using a spectrophotometer ( UV1600, Germany ). The final results were expressed as μM/L. 

## Measurement of NO using the Griess reagent method

The total stable oxidation products of NO metabolism ( NO_2_/NO_3_ ) of seminal plasma were assessed using a Griess reagent. The Griess reagent consists of sulfanilamide ( SULF ) and N-( 1-Naphthyl ) ethylenediaminedihydrochloride ( NEDD ) (23,24). The frozen semen was allowed to thaw and to reach a temperature of 25˚C that was fola lowed by being deproteinized by zinc sulfate solution ( Sigma, America ). The liquefied semen was then centrifuged at 12000 g for 10 minutes. Aliquots ( 300 μL ) of the clear supernatant was mixed with Griess reagents including 300 μL SULF ( 2% w/v, Sigma, America ) in 5% HCl and 300 μL NEDD ( 0.1% w/v, Sigma, America ) in H_2_O in a test tube, while for the reduction of nitrate to nitrite, 300 μL saturated solutions of vanadium ( III ) chloride ( VCl_3_; Sigma, America ) in 1 M HCl was added and incubated for 2 hours at 30˚C in the dark. Then, the absorbance of samples was measured at 540 nm against a blank containing the same concentrations of ingredients but no biological sample. Linear regression was used to determine NO concentration from standard curve of NaNO_2_. The final results were expressed as μmol/L. 

## Statistical analysis

One-way analysis of variance ( ANOVA ) was used to compare NO and TAC levels as well as other parameters including age, volume, morphology and total sperm count among the groups. The data were presented as mean ± standard deviation ( SD ). Percentage motility and sperm count were not normally distributed; therefore, the differences among the groups were done using the Median test ( 25^th^and 75^th^percentiles ). The odds ratios ( OR ) were presented with their 95% confidence intervals ( CI ) using the statistical package for the social sciences ( SPSS; SPSS Inc., Chicago, IL, USA ) version 18. In all cases, a p value of <0.05 was considered statistically significant. 

## Ethical considerations

This study was conducted with the approval of the Medical Research Ethics Committee of the Faculty of Babol University of Medical Sciences, Babol, Iran. An informed written consent was obtained from all subjects participating in the study. 

## Results

After studying thedemographic information and medical history, a total of 95 semen samples were divided into three groups: F.ns ( n=32 ), IF.ns ( n=30 ), including 1 asthenoterato, 1 oligo, 2 astheno, 4 oligoterato, 15 terato and 7 oligoasthenoterato; and IF.s ( n=33 ) including 4 asthenoterato, 4 oligoterato, 18 terato and 7 oligoasthenoterato. 

## Semen analysis

According to the present results, all sperm parameters, including count, motility and morphology, among the three groups of the F.ns, IF.ns and IF.s were strongly significant ( p<0.0001, Table 1 ). But these differences in standard sperm variables between the IF.s and IF.ns were not statistically significant. Semen volume and the mean of age showed no significant difference among the three groups ( p=0.41 and p=0.56, respectively ). 

## TAC and NO assays

Comparison of the TAC and NO levels is shown in table 2. The mean NO levels in the seminal plasma was lower in the F.ns<IF.ns< IF.s, respectively. Also, NO levels showed a significant difference among the three groups ( p=0.018 ). In contrast, the mean TAC levels in the seminal plasma was higher in the F.ns>IF.ns> IF.s, respectively, but these differences were not statistically significant among three groups ( p=0.096, table 2 ). Moreover, TAC and NO levels showed significant differences between F.ns and IF.s groups ( p=0.05 and p=0.01, respectively ). 

**Table 1:** Comparison of sperm parameters in semen samples among the three groups of fertile non-smoking, infertile smoking and non-smoking men


Variables	F.ns (n=32)Mean ± SD	IF.ns (n=30)Mean ± SD	IF.s (n=33)Mean ± SD	P value

**Volume (ml)**	3.79 ± 1.39	3.95± 1.92	3.43± 1.29	0.407
**Sperm morphology (%)**	31.22 ± 14.21	7.40± 5.17	6.67± 4.30	<0.0001
**Total spermcount (×10^6^)**	337.50 ± 140.90	164.83 ± 147.26	155.41 ± 148.72	<0.0001
	Median (25^th^, 75^th^)	Median (25^th^, 75^th^)	Median (25^th^, 75^th^)	
**Sperm count (×10^6^/ml)**	100.0 (82.0, 100.0)	30.0(10.0, 72.5)	40.0(11.0, 70.0)	<0.0001
**Sperm motility (%)**	60.0 (60.0, 70.0)	50.0(37.5, 50.0)	50.0(40.0, 60.0)	<0.0001


F.ns; Fertile non-smokers, IF.ns; Infertile non-smokers, IF.s; Infertile smokers, SD; Standard deviation and n; Number. P< 0.05 was considered statistically significant. One-way analysis of variance (ANOVA) was used for comparisons of volume, morphology and total sperm count among groups. Median test was used for comparisons of sperm count and motility among groups.

**Table 2:** Comparison of TAC and NO concentration of the seminal plasma among the three groups of fertile non-smoking, infertile smoking and non-smoking men


Variables	F.ns (n=33)	IF.ns (n=30)	IF.s (n=32)	P value

**TAC (µM/L)**	2136.25 ± 722.24	1799.17 ± 729	1785.45 ± 718.180	0.096
**NO (μmol/L)**	5.26 ± 3.32	7.26 ± 5.53	8.61 ± 4.96	0.018


Values are expressed as mean ± SD. TAC; Total antioxidant capacity, NO; Nitric oxide, F.ns; Fertile non-smokers, IF.ns; Infertile non-smokers, IF.s; Infertile smokers, SD; Standard deviation and n; Number. P< 0.05 was considered statistically significantusing one-way ANOVA.

## Discussion

The etiology of defective sperm function is closely associated with the over production of ROS. It is important to concentrate on these signaling molecules involved in essential sperm pathways such as capacitation and oxidative damage in spermatozoa (25). In this study, we investigated the relationship between cigarette smoking with NO and TAC levels of seminal plasma to show an increase in oxidative damage level and infertility rate in men. Fraga et al. (26) found the level of 8-hydroxy-2´-deoxyguanosine ( 8OHdG ), a marker of DNA oxidation, is 50% higher in smokers compared with non-smokers ( p=0.005 ). Smoking may also result in chromatin abnormalities and/or endogenous DNA strand breaks in human spermatozoa (27). Several studies have showed that cigarette smoking has an effect on reduced sperm quality ( count, motility and morphology ), especially for those who are heavy smokers or have smoked for many years (5,28,32). In a study from Denmark, of >2500 healthy male participating, a statistically significant inverse dose-response relationship was observed between current cigarette smoking and semen analysis ( semen volume, total sperm count and percentage of sperm motility ). Heavy smokers ( >20 cigarettes per day ) had ~19% lower mean sperm concentration and a 29% lower total sperm count than non-smokers (32). These studies have shown that the decreased sperm quality in smokers can be due to OS. Pasqualotto et al. (31) evaluated 522 F.ns and 367 fertile smokers in three groups based on the mode of smoking doses. They found a decreasing semen volume with an increasing number of cigarettes smoke, but no statistically significant differences were observed among the groups in terms of sperm concentration and motility. Studies have also shown that smokers are more susceptible to OS, leading to adecline in seminal plasma antioxidants such as vitamin E (26) and vitamin C (33). According to a study of 40 smoking and non-smoking male university students ( 22-25 years ) in West Bengal, India, malondialdehyde ( MDA ) level and the lactate dehydrogenase ( LDH ) activity in serum and neutrophil were higher in smokers ( n=20 ) than the non-smokers ( n=20 ). The activities of superoxide dismutase ( SOD ), catalase ( CAT ), glutathione peroxidase ( GPx ), and glutathione reductase ( GR ), as well as glutathione ( GSH ) levels were significantly lower in smokers than the non-smokers in both serum and neutrophil (3). They suggested that the oxidative damage found in smokers could be the result of direct effects of oxidants in cigarette smoke on the decreased antioxidant status (3). High levels of LPO with concomitant depletion of antioxidants in seminal plasma were observed in an Indian population consisting of 100 smokers as compared to 100 non-smokers subjects (34). Moreover, enzymatic and non-enzymatic antioxidants activity was significantly higherin smokers than non-smokers. They have suggested that probably some of the harmful effects of smoking are mediated by free radicals, leading to presence of LPO and breakdown of antioxidant status in cigarette smoking. It suggests that NO may be involved in the etiology of defective sperm function, as well as has a bilateral role, being both a cytotoxic and a necessary molecule for normal sperm production and motility that depends on the alternative redox state and relative NO levels (16,19). A significant linear negative correlation has been reported between NO levels in seminal plasma and sperm motility (13,19,35), morphology (19,35,36) sperm count (19) and viability (35) in infertile men. It has been reported that O_2_, H_2_O_2_and NO can activate target membranes in order to trigger the intracellular mechanisms involved in sperm capacitation. Catalase blocks NO-induced capacitation, but the mechanisms of action involving H_2_O_2_are still unknown. NO may react with O_2_to form ONOO^-^, leading to reaction with glutathione, cysteine, deoxyribose, and other thiols/thioethers (37). This can result in formation of reactive nitrating species in the presence of metal ions or complexes. The dual effects of NO depend on its concentration and reactions with ROS such as H_2_O_2_(37,38).The first mechanism, NO inducing sperm damage, is likely to be involved in inhibition of mitochondrial respiration and DNA synthesis using nitrosylation of heme in mitochondrial enzymes, aconitase, and glyceraldehyde phosphate dehydrogenase that results ina depletion of adenosine triphosphateand the reduction of sperm motility (38). A study in Italy has provided evidence that NO concentration in the seminal plasma of the idiopathic as the nozoospermic infertile patients is significantly higher than those in normozoospermic fertile subjects. Moreover, they showed a significant negative linear correlation between NO concentration and sperm motility, as well as other kinetic parameters of spermatozoa such as VCL and VSL (13). Therefore, they have suggested that the high NO levels and the consequent excessive exposure to OS have a potential pathogenetic role in human sperm motility (13). Sheikh et al. (39) showed higher levels of DNA damage and NO in infertile men compared with fertile men. On the other hand, TAC levels in infertile men were significantly lower than fertile men. Also, a negative correlation was observed between NO concentration and TAC levels in infertile cases ( p<0.001; r=–0.293 ). Amiri et al. (35) demonstrated that mean NO level of seminal plasma in infertile men was significantly higher than fertile donors. Badade et al. (19) investigated seminal levels of NO and TAC in fertile and infertile men. In their study, seminal levels of NO were significantly higher ( p<0.001 ), while TAC was significantly lower ( p<0.001 ) in infertile patients than fertile men. In our previous study, a significant increase in MDA levels and decrease in the mean TAC in the seminal plasma were found in asthenoteratospermic smokers and non-smokers as compared to normozoospermic men, but no significant differences were observed between the smoking and non-smoking patients (30). In the present study, TAC concentration of seminal plasma was higher in F.ns than IF.ns and IF.s ( F.ns>IF.ns>IF.s, respectively ). But these differences were not statistically significant among the three groups ( p= 0.096 ). In contrast, seminal NO levels showed a significant difference among the groups ( p=0.018 ), so it was higher in IF.s than IF.ns and also F.ns ( IF.s>IF.ns>F.ns, respectively). However, the following limitations were applied in our study. Smoking data, as all the other variables in the present study, were collected through self–completed questionnaires, which may have introduced a risk of misclassification of the exposure variable. In addition, the participation rate was low in our study, which may be the reason that we were not able to detect the significant correlation between parameters. Although we included subjects who smoked a minimum of 10 cigarettes per day for at least 2 years, we were unable to classify this group ( IF.s ) based on the number of cigarettes consumed per day and the duration of smoking in years and to examine the relationship between smoking and semen parameters in a dose-dependent fashion because of the subjective nature of the smoking history. The other limitation of our study may be the lack of information regarding the exact etiology of infertility or reproductive potential in these patients. Despite these limitations, our study shows that cigarette smoking and infertility in men are related with increased levels of seminal OS. 

## Conclusion

It is deduced from our results that infertile smoking men have higher levels of seminal OS than fertile and IF.ns. The correlation between cigarette smoking and high OS levels in the seminal plasma can be attributed in part to the associated increase in seminal NO levels and an impaired oxidant defense system. It suggests that smoking induces OS either with the increased levels of oxidants originating from the smoke such as NO or with the decreased levels of antioxidants in the seminal plasma, leading to a decrease in fertilizing potential of spermatozoa in infertile men. 
